# Ensemble test for microbiome data

**DOI:** 10.1186/s40168-026-02367-z

**Published:** 2026-03-10

**Authors:** Deliang Bu, Jingxin Yan, Wanshuo Yang, Xiaoyu Zhang, Qizhai Li

**Affiliations:** 1 School of Statistics, Capital University of Economics and Business, Beijing, 100070 China; 2 State Key Laboratory of Mathematical Sciences, Academy of Mathematics and Systems Science, Chinese Academy of Sciences, Beijing, 100190 China; 3 School of Mathematical Sciences, University of Chinese Academy of Sciences, Beijing, 100049 China; 4 College of Mathematics and Statistics, Chongqing University, Chongqing, 401331 China

**Keywords:** Microbiome data, PERMANOVA, Ensemble test

## Abstract

**Motivation:**

Recent research has revealed strong correlations between the human microbiome and various diseases. However, statistical analysis of microbiome data remains challenging due to its inherent sparsity and high dimensionality. PERMANOVA (Permutational multivariate analysis of variance using distance matrices) has been extensively employed to test the association between microbiome data and biological features. Its non-parametric nature makes it appealing, as it does not impose restrictions on data dimension or distribution. Despite its merits, several limitations have restricted its further application.

**Results:**

This paper introduces E-MANOVA (Ensemble multivariate analysis of variance using distance matrices), a method designed to address these limitations. Traditional PERMANOVA lacks consistent robustness across different distance metrics and association signals, which can lead to power reduction in specific scenarios. Leveraging the idea of ensemble learning, we construct base tests by taking the similarity matrix to the *r*th power and then combine these tests to build a final ensemble test. Our resulting test statistic exhibits high power and robustness compared to other existing methods. Furthermore, we employ direct moment approximation and the Pearson type III distribution to approximate the permutation null distribution, completely avoiding the computationally intensive permutation procedure. Finally, we utilize the Cauchy combination method to aggregate *p*-values from multiple distances, eliminating the need to pre-specify a single distance measure before analysis.

**Conclusions:**

Our extensive simulations demonstrate that the proposed method outperforms existing methods across various situations. Further analysis of real data from cigarette smokers and curated microbiome data shows that our proposed method identifies the highest number of significant associations among all competing methods.

Video Abstract

**Supplementary Information:**

The online version contains supplementary material available at 10.1186/s40168-026-02367-z.

## Introduction

The microbiome is the collection of all microbes, such as bacteria, fungi, viruses, and their genes, that naturally live on and inside human bodies. These microbes outnumber our somatic and germ cells by an estimated 10-fold [1] and provide us with traits we have not had to evolve on our own [2]. Biological and empirical evidence suggests that a considerable part of the environmental influence on human health and disease risk may be mediated or modified by microbial communities [3]. With the development of 16S ribosomal DNA-targeted [4] and whole genome shotgun (WGS) sequencing technology [5], researchers are able to gain deeper insights into the microbiome community and its associations with various human diseases, such as type II diabetes [6], liver cirrhosis [7], atherosclerosis [8] and hypertension [9].

The central goal of human microbiome studies is to identify potential biological and environmental factors that are associated with microbiome composition and to define the relationship between microbiome features and biological or clinical outcomes [10]. However, statistical analyses of microbiome data are challenging due to their complex characteristics. Based on sequence similarity, microbiome data can be clustered into operational taxonomic units (OTUs) and their relative abundances can be measured. These abundance data are compositional (relative abundances sum to one), highly sparse (containing a disproportionally large number of zeros [11]), and high-dimensional (the dimension of relative abundances is often larger than the sample size [12]). These distinct features of microbiome data preclude the application of traditional methods, such as Hotelling’s $T^2$ test.

Numerous methods have been proposed to address the intricate structure of microbiome data, including multivariate two-sample tests [13–15], kernel-based approaches [16, 17] and others [18–21]. Among these methods, PERMANOVA (Permutational multivariate analysis of variance based on distance matrices) [10] stands out as one of the most popular approaches. PERMANOVA aims to identify covariates that can significantly explain the inter-subject variability captured by the pairwise distances. Originally developed in the field of ecology [22], PERMANOVA has found extensive application in testing associations within microbiome data [10, 23, 24]. Its popularity stems from three main advantages. Firstly, as a nonparametric method, PERMANOVA operates without presuming the distribution of relative abundance or the dimensionality of OTUs, even when the dimension surpasses the sample size. Secondly, compared to two-sample tests, PERMANOVA can accommodate not only case-control designs but also continuous covariates, allowing for the incorporation of confounding variables. Thirdly, the distance-based regression framework performs the test based on a similarity matrix, offering flexibility to select similarity measures appropriate for different scenarios.

While PERMANOVA has notable advantages, its limitations have also been widely discussed in the literature [16, 17]. First, as a non-parametric method, it requires permutation to establish its significance, which can be computationally expensive. Second, while the model allows for the selection of multiple distance metrics, determining the most suitable one remains challenging, as non-optimal metrics can reduce power to discover true associations. Tang et al. [24] proposed a method addressing this issue by selecting the minimum *p*-values across various scenarios. However, their approach, utilizing a two-layer permutation for *p*-value calculation, suffers from computational inefficiency. Third, studies have indicated that the asymptotic distribution of the PERMANOVA pseudo-*F* statistic is closely related to a mixture sum of $\chi ^2$ statistics [25]. Notably, the chi-square distribution’s heavy-tailed nature can lead to a loss of power in certain situations when directly summing chi-square distributions.

This paper introduces Ensemble multivariate analysis of variance using distance matrices (E-MANOVA), a method addressing the aforementioned limitations. E-MANOVA, leveraging the idea of ensemble learning, combines the *p*-values of multiple base tests, making it more powerful and robust compared to existing methods. The computational procedure for E-MANOVA utilizes direct moment approximation and Cauchy combination methods, eliminating the need for any permutation procedures, significantly reducing computational time. Our simulations also show that the proposed method achieves greater power than existing association testing methods for microbiome data. Real data analyses of a smoking dataset and curated microbiome data demonstrate that E-MANOVA identifies the most significant associations among all methods tested.

## Methods

### Notations and classic PERMANOVA framework

Suppose we have *n* subjects and *K* represents the number of OTUs. Let $\boldsymbol{X}_1$ be an $n \times p_1$ matrix representing $p_1$ variables of interest, and $\boldsymbol{X}_2$ be an $n \times p_2$ matrix of confounding variables, such as age, gender, and other clinical and environmental variables that are suspected to influence microbial community diversity. Note that all variables are assumed to be centered in this paper. The goal is to test the association between $\boldsymbol{X}_1$ and microbial profiles while adjusting for the confounding variables $\boldsymbol{X}_2$.

Usually, the dimension of OTUs *K* exceeds the sample size *n*, making directly testing the association challenging. The PERMANOVA framework addresses this by first reducing the *K*-dimensional OTU profiles to an $n \times n$ distance matrix, $\boldsymbol{D}=(d_{ij})_{n \times n}$. Following the literature [10, 24, 26], we use the following popular distances for this task. When phylogenetic tree information is available, the weighted UniFrac distance [10] is commonly used. Let $p_{il}$ and $p_{jl}$ denote the proportion of OTUs descending from branch *l* ($l=1, \dots , L$) for samples *i* and *j*, respectively. $b_l$ denotes the length of branch *l*. The weighted UniFrac distance $d_{ij,1}$ is:

$$\begin{aligned} d_{ij,1}=\frac{\sum \nolimits _{l=1}^Lb_l(p_{il}+p_{jl})^{\alpha }|p_{il}-p_{jl}|}{\sum \nolimits _{l=1}^Lb_l(p_{il}+p_{jl})^{\alpha }}, \end{aligned}$$

where $\alpha \in [0,1]$. We use $\alpha =0.5$ as it is considered robust [10]. Another tree-based distance is the unweighted UniFrac distance [24], which only compares presence/absence information:

$$\begin{aligned} d_{ij,2}=\frac{\sum \nolimits _{l=1}^Lb_l|I(p_{il}>0)-I(p_{jl}>0)|}{\sum \nolimits _{l=1}^Lb_l}, \end{aligned}$$

where $I(\cdot )$ is an indicator function. When phylogenetic information is not used, the Bray-Curtis distance [26] is a common choice. Let $p_{ik}$ and $p_{jk}$ be the abundance of OTU *k* ($k=1, \dots , K$) in samples *i* and *j*. The Bray-Curtis distance $d_{ij,3}$ is:

$$\begin{aligned} d_{ij,3}=\frac{\sum \nolimits _{k=1}^K|p_{ik}-p_{jk}|}{\sum \nolimits _{k=1}^K(p_{ik}+p_{jk})}. \end{aligned}$$

Each distance captures different features of the data, and the choice of metric affects the test’s power.

From the chosen distance matrix $\boldsymbol{D}$, a similarity matrix $\boldsymbol{S}=(s_{ij})_{n \times n}$ is then calculated via the transformation $s_{ij}=-\frac{1}{2}d^2_{ij}$. This similarity matrix $\boldsymbol{S}$ is the input for the test statistic. The classic PERMANOVA-based statistic (also known as the pseudo-F statistic), which tests the association between $\boldsymbol{S}$ and $\boldsymbol{X}_1$ while adjusting for $\boldsymbol{X}_2$, is defined as [27, 28]

$$\begin{aligned} T_{0}=\frac{\text {tr}((\boldsymbol{H}_{\boldsymbol{X}}-{\boldsymbol{H}}_{\boldsymbol{X}_2})\boldsymbol{HSH})}{\text {tr}((\boldsymbol{I}_n-{\boldsymbol{H}}_{\boldsymbol{X}}){\boldsymbol{H}}{\boldsymbol{S}}{\boldsymbol{H}})}, \end{aligned}$$

where $\boldsymbol{X} = (\boldsymbol{X}_1, {\boldsymbol{X}}_2)$, $\boldsymbol{H}_{\boldsymbol{X}} = \boldsymbol{X}(\boldsymbol{X}^\top \boldsymbol{X})^{-1}{\boldsymbol{X}}^\top$, $\boldsymbol{H}_{\boldsymbol{X}_2}=\boldsymbol{X}_2(\boldsymbol{X}_2^\top \boldsymbol{X}_2)^{-1}\boldsymbol{X}_2^\top$, and $\boldsymbol{H}=\boldsymbol{I}_n-n^{-1}\boldsymbol{1}_n\boldsymbol{1}_n^\top$ (with $\boldsymbol{1}_n$ being an *n*-dimensional vector of ones). The *p*-value of $T_0$ is typically obtained by permuting the samples. Note that we have eliminated some constants in the definition of $T_0$, as they do not affect the final *p*-value.

### Motivation

Although $T_{0}$ is broadly used, a permutation procedure is needed for calculating its statistical significance [10, 22, 24] and its null distribution is hard to derive. Recently, Shi et al. [25] derived that under the null hypothesis and certain regularity conditions, the numerator term of $T_{0}$, ${\mathrm{ t }r}((\boldsymbol{H}_{\boldsymbol{X}}-{\boldsymbol{H}}_{\boldsymbol{X}_2})\boldsymbol{HSH})$, converges to $\frac{1}{n}\sum \nolimits _{i=1}^{n}\lambda _i\xi _i$ asymptotically and the denominator term converges to some constant as $n \rightarrow +\infty$. Here ${\xi _i} {\mathop {\sim }\limits ^{i.i.d}}\chi _v$, *v* is the rank of $\boldsymbol{H}_{\boldsymbol{X}}-\boldsymbol{H}_{\boldsymbol{X}_2}$ and ${\lambda _i}, i=1,2,\cdots , n$ are the eigenvalues of matrix $\boldsymbol{HSH}$. We can see that the approximate distribution of $\text {tr}((\boldsymbol{H}_{\boldsymbol{X}} - \boldsymbol{H}_{\boldsymbol{X}_2}) \boldsymbol{HSH})$ is a weighted sum of chi-square distributions with the weights determined by the eigenvalues of $\boldsymbol{HSH}$. As it is well known that the chi-square distribution is a heavy-tailed distribution, the power of $T_{0}$ can decrease drastically if a large number of chi-square distributions are directly summed. Thus it is crucial to adjust the weights so that the random variables containing signal information among ${\xi _i}, i=1,2, \cdots , n$ have large weights. However, determining which ${\xi _i}$ contains association signals cannot be done without prior information. Thus, the problem then becomes how to develop powerful tests in various scenarios.

Ensemble learning constitutes a class of robust and efficient machine learning methods, including random forests [29] and bagging [30]. These methods operate on the principle of constructing multiple base learners, which are then aggregated to produce a final, enhanced predictive model. The aggregation process typically involves either majority voting or averaging. While individual base learners may exhibit suboptimal performance, the ensemble method effectively integrates the strengths of these weak learners, resulting in a significant improvement in overall predictive accuracy. Recently, the idea of ensemble has been introduced in testing composite hypotheses [31]. Here we also use the idea of ensemble to improve the power of PERMANOVA. Developing an ensemble procedure for hypothesis testing requires two major steps: constructing some base tests and combining them to obtain a final ensemble test.

### Construction of base tests

To build an individual base test, we note that the weights of the null distribution are directly determined by the eigenvalues of $\boldsymbol{HSH}$; thus, adjustments can be made to $\boldsymbol{HSH}$ to further increase the power of $T_0$. Here we take $\boldsymbol{HSH}$ to the *r*th power so that the eigenvalues of $\boldsymbol{HSH}$ are adjusted. To do this, we need to ensure $\boldsymbol{HSH}$ is positive semi-definite; we use a similar technique as [17]. Denote $\boldsymbol{K} = \boldsymbol{HSH}$. By performing eigenvalue decomposition, $\boldsymbol{K} = \boldsymbol{U \Lambda U}^\top$, where $\boldsymbol{\Lambda } = diag(\lambda _1, \lambda _2, \cdots , \lambda _n)$. Then we reconstruct $\boldsymbol{K}^*$ as $\boldsymbol{K}^* = \boldsymbol{U \Lambda }^* \boldsymbol{U}^\top$ with $\boldsymbol{\Lambda }^*=diag(|\lambda _1|, |\lambda _2|, \cdots , |\lambda _n|)$. Additionally, since the denominator of $T_0$ converges to a constant, we directly omit the denominator term to reduce the computational time. Finally, the individual base statistic $T_{d,r}$ is defined as

$$\begin{aligned} T_{d,r}=\text {tr}((\boldsymbol{H}_{\boldsymbol{X}}-\boldsymbol{H}_{\boldsymbol{X}_2})(\boldsymbol{K}^*)^{r}), r>0, \end{aligned}$$

here $d=1,2,3$ specifies the distance we used, $d=1$ indicates $T_{d,r}$ is calculated with weighted UniFrac distance, $d=2$ indicates unweighted UniFrac distance, and $d=3$ corresponds to Bray-Curtis distance. Compared to the original $T_0$, we raise the matrix $\boldsymbol{HSH}$ to the *r*th power and ignore the denominator which converges to a constant asymptotically. By taking different values of *r*, the eigenvalues $\lambda _i, i=1,2,\cdots , n$ are changed, which in turn affects the weights of the chi-square distribution. Thus the power of $T_{d,r}$ can be increased if the value of *r* is correctly specified under different situations. Also, since different distances focus on different scenarios, the test statistics $T_{d,r}$ with various *d* and *r* will have different performance in different scenarios. It is also notable that when $r=1$, $T_{d,r}$ is analogous to the traditional pseudo-*F* statistic $T_0$ and is expected to have the same power.

### *P*-value computation of base test

Let *D* and *R* represent the sets of selected values for *d* and *r*, respectively, with cardinality denoted by $\mathcal D$ and $\mathcal R$. We now have a total of $\mathcal D \times \mathcal R$ base statistics. A sample-generating permutation procedure can certainly be used to calculate the $\mathcal D \times \mathcal R$ *p*-values of the base tests. However, it is well known that this procedure is computationally expensive, particularly when repeated $\mathcal D \times \mathcal R$ times. Here we propose a new method for calculating the *p*-values using the Pearson type III distribution [32] that can avoid the complex sample-generating procedure and directly calculate the permutation *p*-value. As we prove in Supplemental Material Section 1, we can directly calculate the first three moments of the permutation null distribution of $T_{d,r}$ without generating random samples. Denote the first three moments as $E_p(T_{d,r})$, $Var_p(T_{d,r})$ and $E_p(T_{d,r}^3)$. The detailed formulas are complex and can be found in Supplementary Material *Theorem 1*. We can calculate the skewness of $T_{d,r}$ by

$$\begin{aligned} \gamma _p(T_{d,r})=\frac{E_p(T_{d,r}^3)-3E_p(T_{d,r})Var_p(T_{d,r})-E^3 _p(T_{d,r})}{Var^{\frac{3}{2}}_p(T_{d,r})}. \end{aligned}$$

The method of moments for the Pearson type III distribution can be used to approximate the null distribution of $T_{d,r}$ with the obtained $E_p(T_{d,r})$,$Var_p(T_{d,r})$ and $\gamma _p(T_{d,r})$. Denote $\tilde{T}_{d,r}=(T_{d,r}-E_p(T_{d,r}))/\sqrt{Var_p(T_{d,r})}$, $\hat{b}=4/\gamma _p(T_{d,r})^2$. We can then derive that under the null hypothesis, If $\gamma _p(T_{d,r})>0$,

$$\begin{aligned} \tilde{T}_{d,r} \sim P_{\textrm{III}}(1/\sqrt{\hat{b}}, {\hat{b}}, -\sqrt{\hat{b}}). \end{aligned}$$

If $$\gamma _p(T_{d,r})<0$$,

$$\begin{aligned} \tilde{T}_{d,r} \sim P_{\textrm{III}}(-1/\sqrt{\hat{b}}, {\hat{b}}, \sqrt{\hat{b}}). \end{aligned}$$

Here $P_{\textrm{III}}(a,b,c)$ denotes the Pearson type III distribution with scale, shape, and location parameters *a*, *b*, and *c*, respectively. The *p*-value of this distribution can be directly calculated based on the probability of the Pearson type III distribution exceeding the observed value. It should be noted that the Pearson type III distribution is a generalization of the Gamma distribution; here we choose the Pearson type III distribution over the Gamma distribution mainly because the Pearson type III distribution allows for negative skewness, which is commonly encountered in $T_{d,r}$ with $r<0.5$. In Supplementary Material Section 2, we will demonstrate the efficacy of the Pearson type III approximation for the null distribution $T_{d,r}$ through simulations and quantile-quantile plots.

### Combination of base tests

Denote the calculated *p*-values of $T_{d,r}$ as $p_{d,r}$. We still have to combine these $\mathcal D\times \mathcal R$ *p*-values to construct robust test statistics. To achieve this, we employ the Cauchy combination method, which provides an analytical *p*-value without using bootstrap procedures [33]. The final test statistic *T*, named as Ensemble multivariate analysis of variance using distance matrices (E-MANOVA), is calculated as follows

$$\begin{aligned} T=\sum \limits _{d\in D}\sum \limits _{r \in R}\frac{1}{\mathcal {D} \times \mathcal {R}}\hbox {tan}\{(0.5-p_{d,r})\pi \}. \end{aligned}$$

*T* follows a standard Cauchy distribution under the null. Its *p*-value can be easily calculated by

$$\begin{aligned} P=0.5-\{\text {arctan}(T)\}/\pi . \end{aligned}$$

It should be mentioned that the final *p*-value of E-MANOVA can be calculated without any sample generating procedure, which saves a significant amount of time compared to traditional methods.

One important practical issue of E-MANOVA is the choice of the set *R*. Based on empirical selection, we recommend $R=\{0.125,0.25,0.5,1,2\}$. Values below 0.125 or greater than 2 do not lead to a significant change in power and may cause approximation inaccuracy.

## Simulation

### Simulation strategy

Our simulation strategy generally proceeds in two stages. First, microbiome abundance data are generated based on an empirical dataset. Second, continuous or binary outcome variables are computed to represent different scenarios.

The empirical basis was selected from a real-world dataset. We utilized the recently developed *MIDASim* package [34] to generate simulated microbiome data, based on the type I diabetes data [35] found in the R package *curatedMetagenomicData* [36]. This source dataset comprises 89 samples and 330 OTUs. Subsequently, simulated OTUs were generated based on these empirical data, with the sample size set to $n=100$.

**Fig. 1:**
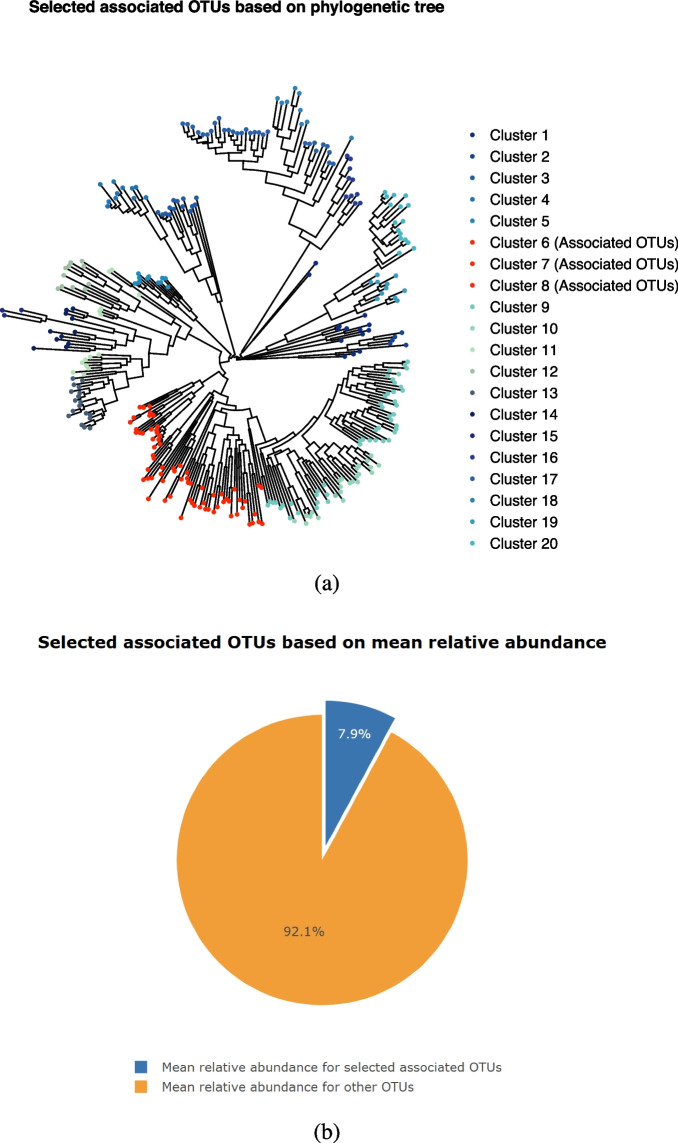
Two different ways for selecting OTUs that associated with outcome variable, illustration with type I diabetes data [35]. **a** The associated OTUs are selected based on phylogenetic tree information, corresponding to scenarios *S*1 and *S*3. **b** The associated OTUs are selected based on mean relative abundance, corresponding to scenarios *S*2 and *S*4

After generating the simulated microbiome abundance data, we designed simulation settings to mimic two common analytical situations. The first situation (Scenarios S1 and S3) incorporates phylogenetic tree information. Here, OTUs associated with the outcome variable are selected based on the phylogenetic tree. To be more specific, we first grouped all OTUs into 20 clusters based on phylogenetic tree distance. Then, we selected several of these clusters to be associated with the outcome variable. Conversely, the second situation (Scenarios *S*2 and *S*4) disregarded phylogenetic information. Instead, we selected OTUs associated with the outcomes solely based on mean relative abundance. To provide a more intuitive demonstration of our simulation strategy, Fig. 1 visually presents the associated OTUs selected using these two distinct approaches. As illustrated in Fig. 1a, corresponding to scenarios *S*1 and *S*3, OTU selection is performed based on the phylogenetic tree. Conversely, Fig. 1b, corresponding to scenarios *S*2 and *S*4, demonstrates OTU selection according to mean relative abundance criteria.

Let $Z_{ij}$ denote the abundance of the *j*th OTU in the *i*th sample, and let *A* be the set of selected OTUs associated with the outcome.

The continuous outcome $y_i$ for sample *i* was generated using the following model:

1 $$\begin{aligned} y_i = 0.5X_{1i} + 0.5X_{2i} + \beta \cdot f\left( \text {scale}\left( \sum \limits _{k \in A} Z_{ik}\right) \right) + \epsilon _i, \end{aligned}$$

where $\text {scale}(\cdot )$ denotes a standardization function (e.g., to zero mean and unit variance) applied to the summed abundance of the selected OTUs. The function $f(\cdot )$ defines the nature of the association between the aggregate OTU signal and the outcome. We considered three distinct relationships distributed across our scenarios:

- **Linear (S1 and S2):** $$f(x) = x$$
- **Nonlinear - Diminishing Effect (S3):** $f(x) = \root 3 \of {x}$
- **Nonlinear - Periodic Effect (S4):** $f(x) = \sin (x)$

In this model, $\epsilon _i$ is a standard normal random variable. The parameter $\beta$ measures the association strength and was adjusted to ensure all methods had comparable statistical power. To account for various covariates, $X_{1i}$ was simulated as a Bernoulli random variable with a success probability of 0.5, and $X_{2i}$ was simulated as a continuous variable that was either independent of or correlated with the OTU data.

For binary outcomes, data were generated under the following logistic regression model:

2 $$\begin{aligned} {\text {logit}}(p_i) = 0.5X_{1i} + 0.5X_{2i} + \beta \cdot f\left( \text {scale}\left( \sum \limits _{k \in A} Z_{ik}\right) \right) , \end{aligned}$$

where $p_i = P(y_i = 1 | X_i, Z_i)$ and the association function $f(\cdot )$ utilized the same linear (S1/S2) and nonlinear (S3/S4) forms as defined for the continuous model.

Finally, we evaluated the performance of our proposed method, E-MANOVA, against a suite of existing and robust methods. The competitors included classic approaches (MIRKAT [17], MISPU [21], and PERMANOVA-S [24], denoted as P-S) as well as recently proposed methods (MIATDS [18], MIHC [19], and RFtest [20]). All comparisons were conducted at a significance level of $\alpha = 0.05$ over 1000 simulation replications.

### Simulation results

The main text presents the simulation results for continuous phenotypes. Results for binary phenotypes were highly similar and are consequently shown in Supplementary Material Section 3.

Table 1 presents the empirical type I error rates for continuous phenotypes of E-MANOVA, MIATDS, MiHC, MiRKAT, MiSPU, P-S, and RFtest at a significance level of $\alpha = 0.05$. As shown, all methods generally controlled the Type I error rate acceptably around the nominal 0.05 level. For instance, under Scenario S1 with independent covariates, the empirical type I error rates of E-MANOVA, MIATDS, MiHC, MiRKAT, MiSPU, P-S, and RFtest were 0.051, 0.053, 0.050, 0.050, 0.047, 0.043, and 0.035, respectively. We observed that the empirical type I error rates for E-MANOVA were occasionally slightly inflated. This is a known characteristic of the Cauchy combination method used for *p*-value aggregation. As noted in the original literature [37], this method can lead to a slight inflation at larger $\alpha$ levels (e.g., 0.05) when *p*-values are correlated, though it maintains high accuracy for smaller *p*-values typically required after multiple testing correction.

**Table 1:** Type I error rates of continuous phenotypes with significance level $\alpha =0.05$

		E-MANOVA	MIATDS	MiHC	MiRKAT	MiSPU	P-S	RFtest
S1, independent covariates	$\alpha =0.05$	0.051	0.053	0.050	0.050	0.047	0.043	0.035
S1, correlated covariates	$\alpha =0.05$	0.056	0.043	0.048	0.061	0.047	0.058	0.051
S2, independent covariates	$\alpha =0.05$	0.057	0.058	0.061	0.041	0.051	0.039	0.054
S2, correlated covariates	$\alpha =0.05$	0.054	0.059	0.058	0.043	0.044	0.039	0.057
S3, independent covariates	$\alpha =0.05$	0.052	0.044	0.037	0.044	0.033	0.045	0.031
S3, correlated covariates	$\alpha =0.05$	0.061	0.036	0.033	0.050	0.054	0.053	0.046
S4, independent covariates	$\alpha =0.05$	0.057	0.058	0.061	0.041	0.051	0.039	0.057
S4, correlated covariates	$\alpha =0.05$	0.054	0.059	0.058	0.043	0.044	0.039	0.057

**Fig. 2:**
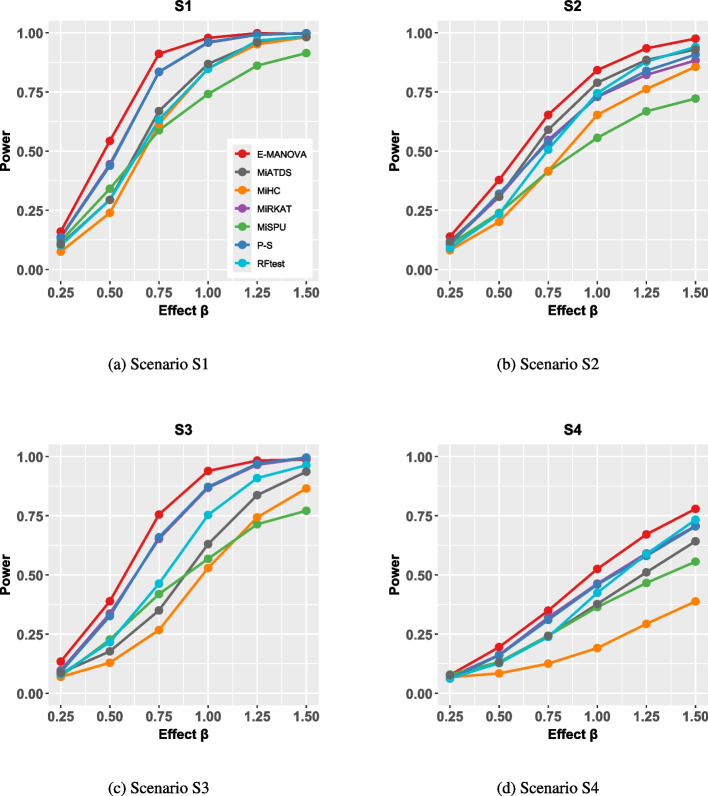
Empirical powers of E-MANOVA, MIATDS, MiHC, MiRKAT, MiSPU, P-S, and RFtest with continuous phenotypes and independent covariates at significance level $\alpha =0.05$

Figure 2 illustrates the empirical power of E-MANOVA, MIATDS, MiHC, MiRKAT, MiSPU, P-S, and RFtest for continuous phenotypes with independent covariates at a significance level of $\alpha =0.05$. The results for binary phenotypes are similar and can be found in Supplementary Material Section 3. From the figure, we can conclude that E-MANOVA consistently demonstrates either superior power or comparable performance to the competing methods.

For example, under the linear scenario S1 (Fig. 2a) at an effect size of $\beta =0.50$, E-MANOVA achieved a power of 0.54, while the strongest competitor (MiRKAT) reached 0.45 (a power increment of 0.09). This advantage also held in nonlinear settings. In scenario S3 (diminishing effect, Fig. 2c) with $\beta =0.75$, E-MANOVA’s power was 0.76, surpassing the next best method (P-S) at 0.66 (a power increment of 0.10).Similarly, in scenario S4 (periodic effect, Fig. 2d) with $\beta =1.25$, E-MANOVA’s power was 0.67, compared to 0.59 for the strongest competitor (MiRKAT).The robust performance of E-MANOVA across these diverse conditions stems from its aggregation of *p*-values derived from multiple distance metrics and *r* values. As individual distance metrics can exhibit varying performance across scenarios, this multi-distance, combined approach is necessary for robust power.

Finally, we observed that when the covariates are correlated with the associated OTU, the power of all tests decreased, as expected. For example, in scenario *S*2, effect size $\beta =1$, under independent covariates, the power of E-MANOVA, MIATDS, MiHC, MiRKAT, MiSPU, P-S, and RFtest was 0.84, 0.79, 0.65, 0.73, 0.56, 0.73, and 0.75, respectively. When the covariates were set to be correlated to the associated OTU, the power of the aforementioned methods dropped to 0.65, 0.57, 0.41, 0.52, 0.40, 0.53, and 0.51, respectively. These facts indicate the challenge that correlated covariates pose for microbiome-based association tests.

### Computational time

E-MANOVA not only demonstrates robustness and high statistical power in microbiome analysis but also exhibits notable computational efficiency. This section presents a comparative analysis of the computational performance of E-MANOVA against two PERMANOVA-based methods: P-W (traditional PERMANOVA with weighted UniFrac distance) and P-S. To ensure a fair comparison, all methods were implemented entirely in R and executed on a laptop equipped with an Intel Core i9-13900H CPU.

Table 2 presents the computation time of the three methods, where *n* stands for the sample size analyzed and *B* stands for the number of permutations used. A large *B* is essential for generating *p*-values reaching stringent significant levels, as a permutation-based method cannot generate *p*-values smaller than 1/*B*.

**Table 2:** Computational times of P-W, P-S, and E-MANOVA

	Method	*n* = 100	*n* = 200	*n* = 500
$B=1000$	P-W	3.23 s	24.85 s	348.61 s
	P-S	10.32 s	72.65 s	1046.98 s
	E-MANOVA	0.23 s	1.61 s	18.48 s
$B=2000$	P-W	6.89 s	44.74 s	981.87 s
	P-S	22.88 s	125.11 s	1960.85 s
	E-MANOVA	0.23 s	1.45 s	8.57 s
$B=5000$	P-W	17.74 s	117.63 s	1746.03 s
	P-S	52.40 s	357.36 s	5677.48 s
	E-MANOVA	0.26 s	1.48 s	19.79 s

*B* stands for number of permutation and *n* stands for number of samples analyzed

From the table, we can see that our proposed method computes much more efficiently than other methods. The larger the *n* and *B*, the more obvious the advantage. With $n=100$ and $B=1000$, the computational time of P-W, P-S, and E-MANOVA was 3.23 s, 10.32 s, and 0.23 s, respectively. This disparity escalates dramatically as *n* and *B* grow. With $n=500$ and $B=5000$, the computational time of P-W and P-S reached 1746.03 s and 5677.48 s, which is computationally prohibitive for many practical applications. In contrast, the computational time of E-MANOVA was merely 19.79 s. The main reason for this superior performance is that E-MANOVA computes *p*-values analytically via a direct approximation method and the Cauchy combination method, which avoids the computationally expensive process of permutation completely.

A second, crucial advantage is the deterministic nature of our method. E-MANOVA does not rely on any sample generation to calculate its *p*-value. In contrast, P-W, P-S, and other methods like MiRKAT and MiSPU, rely on the generation of random samples to calculate their *p*-values. Consequently, multiple runs of these methods may yield varying results. This inconsistency might lead to results being inconclusive, particularly for *p*-values near the significance threshold. Conversely, with E-MANOVA, performing the method multiple times using the same dataset consistently produces identical results.

## Real data analysis

### Application to smoke data

Cigarette smokers have an increased risk of multiple diseases, including upper respiratory tract infections. Charlson et al. [12] conducted an investigation of the smoking effect on the oropharyngeal and nasopharyngeal microbiome. This data has been broadly used as a benchmark dataset for microbiome data analysis [10, 15–17]. After data preprocessing, the data consists of 60 samples (28 smokers and 32 non-smokers) and 856 OTUs. Our main goal is to test the association between smoking status and OTU data. Other variables including sex and age can be seen as potential confounding variables. Phylogenetic tree information is also included in the dataset. This dataset can be publicly downloaded from R package *GUniFrac*.

We start by conducting the association test without adjusting any confounding variables, following the original study [10]. Seven methods were applied to test for the association including E-MANOVA, MIATDS, MiHC, MIRKAT, MISPU, P-S, and RFtest. The *p*-values of these seven methods were $1.54 \times 10^{-3}$, $1.06 \times 10^{-2}$, $8.24 \times 10^{-2}$, $3.07\times 10^{-3}$, $7.20\times 10^{-3}$, $6.60\times 10^{-3}$, and $2.00\times 10^{-3}$, respectively. All 7 methods yielded significant results under significance level 0.05. This result is consistent with all previous analyses [10, 17]. Notably, our proposed method E-MANOVA generated the most significant result with a *p*-value of $1.54 \times 10^{-3}$. This *p*-value is also smaller than the smallest *p*-value 0.006 reported in the original study [10].

In a subsequent study, Zhao et al. [17] argued that the imbalance in the proportion of male and female subjects indicates strong potential for confounding (75% smokers were male while only 56% non-smokers were male): it was unclear whether the differences in microbiome profiles between smokers and non-smokers are driven by smoking or by the gender imbalance. Thus, here we consider sex as a confounding variable and test the association between microbiome data and smoking status while adjusting for the sex variable. The *p*-values of the aforementioned seven methods were re-calculated. The new *p*-values for these seven methods were $4.49 \times 10^{-3}$, $2.63 \times 10^{-2}$, $3.10 \times 10^{-1}$, $1.17\times 10^{-2}$, $8.90\times 10^{-3}$, $8.60\times 10^{-3}$, and $5.00\times 10^{-3}$. The *p*-values for all methods changed after adjustment, indicating that sex is indeed a confounding variable. Again, the *p*-value of E-MANOVA ($4.49 \times 10^{-3}$) was the smallest among all competing methods.

### Application to curated microbiome data

In this section, we analyze recently proposed microbiome data contained in the R package *curatedMetagenomicData*, which provides uniformly processed human microbiome data from different studies [36] and has been broadly used by various recently proposed microbiome-based methods [38–40]. Version 3.14 of the package, used in our paper, contains 22,588 samples collected from 93 studies with various diseases including type 2 diabetes, colorectal cancer, and inflammatory bowel disease.

We first analyzed the case-control data contained in the package. Since the original data originates from different studies, during the data preprocessing procedure, we removed studies based on the following criteria: (1) studies lacking control groups, (2) studies with fewer than 10 samples in either the control or case group, and (3) studies with excessively complex data structures. After data preprocessing, we obtained a total of 37 datasets from 30 studies spanning diseases including: Parkinson’s disease (PD), schizophrenia, Alzheimer’s disease (AD), colorectal cancer (CRC), adenoma, mucositis, peri-implantitis, inflammatory bowel disease (IBD), type 1 diabetes (T1D), atherosclerotic cardiovascular disease (ACVD), impaired glucose tolerance (IGT), type 2 diabetes (T2D), hypertension, myalgic encephalomyelitis/chronic fatigue syndrome (ME/CFS), soil-transmitted helminth (STH), *Clostridium difficile* infection (CDI), asthma, migraine, and bipolar disorder (BD). To avoid cross-study differences that may lead to false positives, if two studies analyzed the same disease, we treated them as separate datasets. Additionally, for a single study involving multiple diseases, we treated each disease as an independent dataset sharing the same control group. Detailed information on the 37 datasets can be found in Table 3. We then applied all seven methods (E-MANOVA, MIATDS, MiHC, MiRKAT, MiSPU, P-S, and RFtest) to the 37 datasets, with the *p*-values presented in Table 4. To control for multiple testing, we applied the Bonferroni correction and set the significance level to $\alpha = 0.05/37 \approx 1.35 \times 10^{-3}$.

**Table 3:** Detailed dataset information

Number	Study	Disease	Total samples	Disease count
1	$\text {BedarfJR\_2017}$ [41]	PD	59	31
2	$\text {Castro-NallarE\_2015}$ [42]	Schizophrenia	28	12
3	$\text {ChngKR\_2016}$ [43]	AD	40	10
4	$\text {FengQ\_2015}$ [44]	CRC	29	13
5	$\text {GhensiP\_2019}$ [45]	Mucositis	84	19
6	$\text {GhensiP\_2019}$ [45]	Peri-implantitis	85	20
7	$\text {GuptaA\_2019}$ [46]	CRC	60	30
8	$\text {HallAB\_2017}$ [47]	IBD	259	185
9	$\text {HanniganGD\_2017}$ [48]	Adenoma	54	26
10	$\text {HanniganGD\_2017}$ [48]	CRC	55	27
11	$\text {HeitzBuschartA\_2016}$ [49]	T1D	45	24
12	$\text {IjazUZ\_2017}$ [50]	IBD	94	56
13	$\text {JieZ\_2017}$ [51]	ACVD	385	214
14	$\text {KarlssonFH\_2013}$ [52]	IGT	92	49
15	$\text {KarlssonFH\_2013}$ [52]	T2D	96	53
16	$\text {KosticAD\_2015}$ [35]	T1D	120	31
17	$\text {LiJ\_2014}$ [53]	IBD	113	103
18	$\text {LiJ\_2014}$ [53]	T1D	41	31
19	$\text {LiJ\_2014}$ [53]	T2D	89	79
20	$\text {LiJ\_2017}$ [54]	Hypertension	196	155
21	$\text {NagySzakalD\_2017}$ [55]	ME/CFS	100	50
22	$\text {NielsenHB\_2014}$ [56]	IBD	396	148
23	$\text {QinJ\_2012}$ [6]	T2D	363	170
24	$\text {RubelMA\_2020}$ [57]	STH	175	89
25	$\text {SankaranarayananK\_2015}$ [58]	T2D	37	19
26	$\text {ThomasAM\_2018b}$ [59]	CRC	60	32
27	$\text {ThomasAM\_2019\_c}$ [59]	CRC	80	40
28	$\text {VincentC\_2016}$ [60]	CDI	196	14
29	$\text {VogtmannE\_2016}$ [61]	CRC	110	52
30	$\text {WirbelJ\_2018}$ [62]	CRC	125	60
31	$\text {XieH\_2016}$ [63]	Asthma	193	24
32	$\text {XieH\_2016}$ [63]	Migraine	205	36
33	$\text {YeZ\_2018}$ [64]	BD	65	20
34	$\text {YuJ\_2015}$ [65]	CRC	83	45
35	$\text {ZellerG\_2014}$ [66]	CRC	114	53
36	$\text {ZellerG\_2014}$ [66]	Adenoma	103	42
37	$\text {ZhuF\_2020}$ [67]	schizophrenia	171	90

Disease refers to the name of the disease under investigation, total samples indicates the total number of samples, and disease count represents the sample size of diseases in the dataset

**Table 4:** Analysis results for different methods

Number	E-MANOVA	MIATDS	MiHC	MiRKAT	MISPU	P-S	RFtest
1	$2.12 \times 10^{-3}$	$3.05 \times 10^{-2}$	$8.16 \times 10^{-2}$	$6.80 \times 10^{-3}$	$1.05 \times 10^{-1}$	$5.45 \times 10^{-3}$	$1.79 \times 10^{-2}$
2	$5.29 \times 10^{-4}$	$1.17 \times 10^{-2}$	$2.69 \times 10^{-2}$	$1.20 \times 10^{-3}$	$7.10 \times 10^{-3}$	$1.25 \times 10^{-3}$	$1.00 \times 10^{0}$
3	$1.11 \times 10^{-3}$	$3.63 \times 10^{-2}$	$1.52 \times 10^{-1}$	$1.67 \times 10^{-2}$	$1.01 \times 10^{-1}$	$2.52 \times 10^{-2}$	$5.00 \times 10^{-4}$
4	$1.91 \times 10^{-7}$	$5.00 \times 10^{-5}$	$9.95 \times 10^{-3}$	$7.50 \times 10^{-5}$	$9.00 \times 10^{-4}$	$5.00 \times 10^{-5}$	$1.50 \times 10^{-4}$
**5**	$\mathbf {1.02 \times 10^{-3}}$	$1.63 \times 10^{-2}$	$8.55 \times 10^{-3}$	$2.13 \times 10^{-3}$	$4.21 \times 10^{-2}$	$1.40 \times 10^{-3}$	$2.15 \times 10^{-2}$
6	$4.56 \times 10^{-14}$	$5.00 \times 10^{-5}$	$5.00 \times 10^{-7}$	$5.00 \times 10^{-5}$	$5.00 \times 10^{-5}$	$5.00 \times 10^{-5}$	$5.00 \times 10^{-5}$
7	$3.28 \times 10^{-14}$	$5.00 \times 10^{-5}$	$5.00 \times 10^{-7}$	$5.00 \times 10^{-5}$	$5.00 \times 10^{-5}$	$5.00 \times 10^{-5}$	$5.00 \times 10^{-5}$
8	$<1 \times 10^{-14}$	$5.00 \times 10^{-5}$	$5.00 \times 10^{-7}$	$5.00 \times 10^{-5}$	$5.00 \times 10^{-5}$	$5.00 \times 10^{-5}$	$5.00 \times 10^{-5}$
9	$5.19 \times 10^{-1}$	$5.28 \times 10^{-1}$	$2.55 \times 10^{-1}$	$6.32 \times 10^{-1}$	$8.32 \times 10^{-1}$	$7.93 \times 10^{-1}$	$3.14 \times 10^{-1}$
10	$7.40 \times 10^{-1}$	$9.18 \times 10^{-1}$	$6.15 \times 10^{-1}$	$8.47 \times 10^{-1}$	$9.74 \times 10^{-1}$	$9.43 \times 10^{-1}$	$2.52 \times 10^{-1}$
11	$4.98 \times 10^{-4}$	$5.10 \times 10^{-3}$	$4.05 \times 10^{-3}$	$1.97 \times 10^{-2}$	$2.60 \times 10^{-2}$	$1.85 \times 10^{-2}$	$1.00 \times 10^{-4}$
12	$<1 \times 10^{-14}$	$5.00 \times 10^{-5}$	$5.00 \times 10^{-7}$	$5.00 \times 10^{-5}$	$5.00 \times 10^{-5}$	$5.00 \times 10^{-5}$	$5.00 \times 10^{-5}$
13	$<1 \times 10^{-14}$	$5.00 \times 10^{-5}$	$5.00 \times 10^{-7}$	$5.00 \times 10^{-5}$	$5.00 \times 10^{-5}$	$5.00 \times 10^{-5}$	$5.00 \times 10^{-5}$
14	$1.12 \times 10^{-2}$	$1.34 \times 10^{-2}$	$1.10 \times 10^{-2}$	$9.70 \times 10^{-3}$	$4.63 \times 10^{-2}$	$1.16 \times 10^{-2}$	$1.22 \times 10^{-2}$
15	$4.24 \times 10^{-4}$	$7.70 \times 10^{-3}$	$2.13 \times 10^{-2}$	$2.45 \times 10^{-3}$	$1.97 \times 10^{-2}$	$1.55 \times 10^{-3}$	$5.00 \times 10^{-5}$
16	$2.07 \times 10^{-13}$	$5.00 \times 10^{-5}$	$5.05 \times 10^{-5}$	$2.00 \times 10^{-3}$	$7.05 \times 10^{-3}$	$2.30 \times 10^{-3}$	$5.00 \times 10^{-5}$
17	$7.77 \times 10^{-6}$	$2.56 \times 10^{-2}$	$1.78 \times 10^{-2}$	$1.33 \times 10^{-3}$	$4.41 \times 10^{-2}$	$2.45 \times 10^{-3}$	$1.30 \times 10^{-3}$
18	$1.29 \times 10^{-6}$	$5.15 \times 10^{-3}$	$1.45 \times 10^{-3}$	$5.50 \times 10^{-4}$	$4.00 \times 10^{-3}$	$3.50 \times 10^{-4}$	$5.00 \times 10^{-5}$
**19**	$\mathbf {4.10 \times 10^{-4}}$	$1.61 \times 10^{-1}$	$1.07 \times 10^{-1}$	$2.35 \times 10^{-3}$	$4.90 \times 10^{-2}$	$3.20 \times 10^{-3}$	$7.55 \times 10^{-3}$
20	$1.43 \times 10^{-1}$	$4.83 \times 10^{-2}$	$4.03 \times 10^{-2}$	$1.65 \times 10^{-1}$	$3.15 \times 10^{-1}$	$2.03 \times 10^{-1}$	$9.95 \times 10^{-2}$
21	$2.20 \times 10^{-6}$	$1.75 \times 10^{-3}$	$9.51 \times 10^{-4}$	$1.00 \times 10^{-4}$	$5.00 \times 10^{-5}$	$5.00 \times 10^{-5}$	$5.00 \times 10^{-5}$
22	$<1 \times 10^{-14}$	$5.00 \times 10^{-5}$	$5.00 \times 10^{-7}$	$5.00 \times 10^{-5}$	$5.00 \times 10^{-5}$	$5.00 \times 10^{-5}$	$5.00 \times 10^{-5}$
23	$<1 \times 10^{-14}$	$5.00 \times 10^{-5}$	$5.00 \times 10^{-7}$	$5.00 \times 10^{-5}$	$3.00 \times 10^{-4}$	$5.00 \times 10^{-5}$	$5.00 \times 10^{-5}$
24	$<1 \times 10^{-14}$	$5.00 \times 10^{-5}$	$5.00 \times 10^{-7}$	$5.00 \times 10^{-5}$	$5.00 \times 10^{-5}$	$5.00 \times 10^{-5}$	$5.00 \times 10^{-5}$
25	$1.20 \times 10^{-1}$	$2.83 \times 10^{-1}$	$2.26 \times 10^{-1}$	$9.40 \times 10^{-2}$	$3.61 \times 10^{-2}$	$1.32 \times 10^{-1}$	$1.08 \times 10^{-1}$
26	$7.57 \times 10^{-7}$	$3.37 \times 10^{-2}$	$4.59 \times 10^{-2}$	$4.00 \times 10^{-4}$	$2.00 \times 10^{-4}$	$2.00 \times 10^{-4}$	$1.80 \times 10^{-3}$
27	$6.90 \times 10^{-4}$	$5.00 \times 10^{-5}$	$2.32 \times 10^{-2}$	$9.75 \times 10^{-3}$	$7.00 \times 10^{-4}$	$8.45 \times 10^{-3}$	$1.00 \times 10^{-4}$
28	$6.43 \times 10^{-8}$	$5.00 \times 10^{-5}$	$5.00 \times 10^{-7}$	$1.00 \times 10^{-4}$	$1.81 \times 10^{-2}$	$5.00 \times 10^{-5}$	$5.00 \times 10^{-5}$
29	$1.34 \times 10^{-2}$	$2.45 \times 10^{-3}$	$1.70 \times 10^{-3}$	$4.69 \times 10^{-2}$	$7.34 \times 10^{-2}$	$5.32 \times 10^{-2}$	$1.44 \times 10^{-2}$
30	$<1 \times 10^{-14}$	$5.00 \times 10^{-5}$	$3.51 \times 10^{-4}$	$7.50 \times 10^{-5}$	$5.00 \times 10^{-5}$	$5.00 \times 10^{-5}$	$5.00 \times 10^{-5}$
31	$2.19 \times 10^{-2}$	$8.35 \times 10^{-3}$	$4.20 \times 10^{-3}$	$1.85 \times 10^{-2}$	$9.35 \times 10^{-2}$	$2.10 \times 10^{-2}$	$1.29 \times 10^{-1}$
32	$2.15 \times 10^{-2}$	$1.70 \times 10^{-3}$	$9.51 \times 10^{-4}$	$1.06 \times 10^{-2}$	$2.95 \times 10^{-2}$	$1.50 \times 10^{-2}$	$3.10 \times 10^{-3}$
33	$1.06 \times 10^{-1}$	$9.87 \times 10^{-2}$	$1.86 \times 10^{-1}$	$1.38 \times 10^{-1}$	$2.35 \times 10^{-1}$	$1.50 \times 10^{-1}$	$2.98 \times 10^{-2}$
34	$2.80 \times 10^{-10}$	$2.40 \times 10^{-2}$	$2.02 \times 10^{-1}$	$7.50 \times 10^{-5}$	$5.00 \times 10^{-5}$	$5.00 \times 10^{-5}$	$5.00 \times 10^{-5}$
35	$3.97 \times 10^{-9}$	$5.00 \times 10^{-5}$	$5.00 \times 10^{-7}$	$7.50 \times 10^{-5}$	$1.03 \times 10^{-2}$	$5.00 \times 10^{-5}$	$5.00 \times 10^{-5}$
36	$9.79 \times 10^{-4}$	$1.95 \times 10^{-1}$	$3.32 \times 10^{-1}$	$9.50 \times 10^{-4}$	$3.71 \times 10^{-1}$	$2.00 \times 10^{-3}$	$1.59 \times 10^{-1}$
37	$3.04 \times 10^{-13}$	$1.25 \times 10^{-3}$	$5.01 \times 10^{-4}$	$1.00 \times 10^{-4}$	$5.00 \times 10^{-5}$	$5.00 \times 10^{-5}$	$1.00 \times 10^{0}$

This table presents the *p*-values of E-MANOVA, MIATDS, MiHC, MiRKAT, MISPU, P-S, and RFtest applied on different datasets. The datasets that only detect significant by E-MANOVA are marked in bold face

**Fig. 3:**
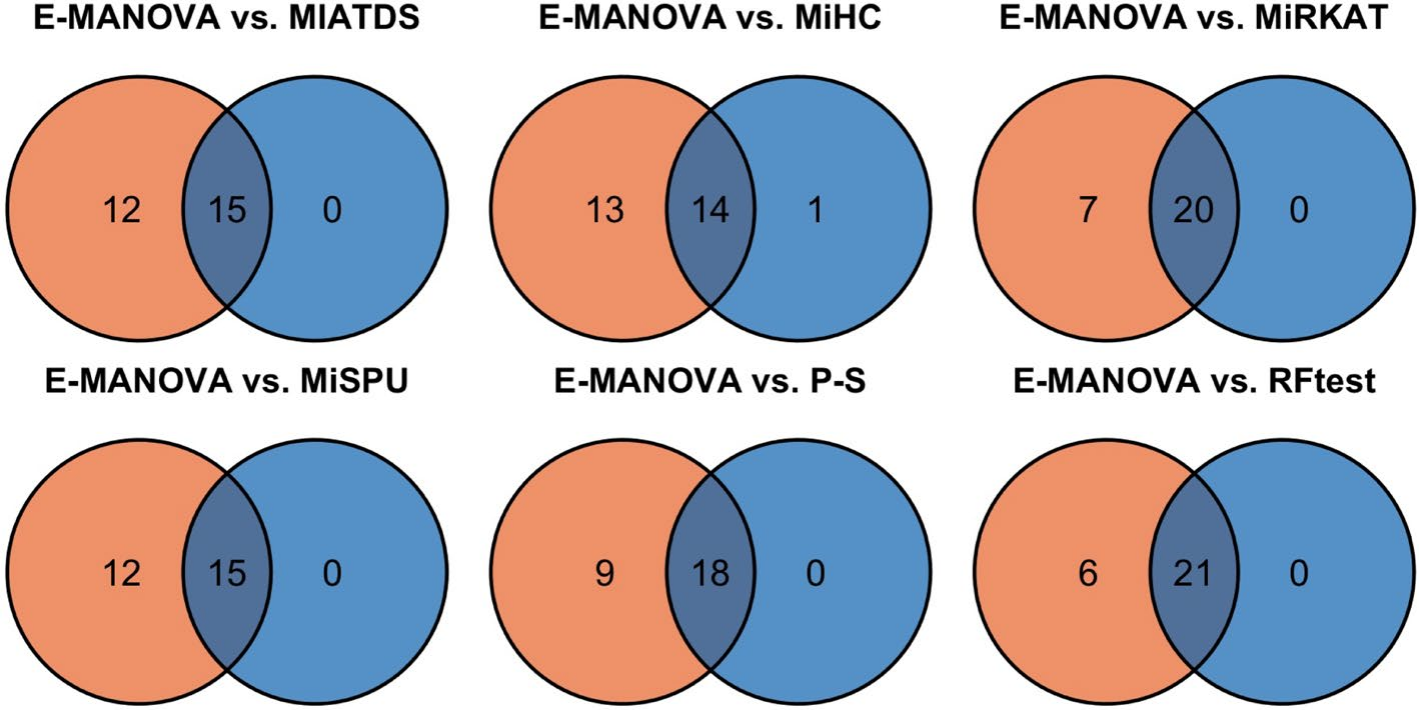
Venn diagrams illustrating the overlap in significant datasets detected by E-MANOVA versus six competing methods. Each panel shows the number of datasets uniquely identified by E-MANOVA (orange), uniquely by the competitor (blue), and jointly by both. The significance level was set at $\alpha = 0.05/37 \approx 1.35 \times 10^{-3}$

Figure 3 presents a series of Venn diagrams illustrating the pairwise overlap of datasets identified as significant between E-MANOVA and each of the six competing methods. A clear pattern emerged from these comparisons: our proposed method, E-MANOVA, identified 27 significant datasets, a number substantially higher than any other method. This indicates that our proposed method possesses greater statistical power to detect associations between the microbiome and various diseases. Notably, 2 of the 37 datasets were found to be significant exclusively by our method. These datasets correspond to Mucositis and T2D, associations that have been reported in other literature [68, 69].

The *curatedMetagenomicData* package also contains various continuous phenotypes. Here, we selected three crucial indicators for human health: body mass index (BMI), systolic blood pressure (SBP), and diastolic blood pressure (DBP). We then tested the association of these three indicators with microbiome data using the seven aforementioned methods. To avoid cross-study differences, the BMI data ($n=45$) was sourced from [41], while the SBP and DBP data ($n=117$) were collected from [70].

The resulting *p*-values for BMI obtained from E-MANOVA, MIATDS, MiHC, MiRKAT, MiSPU, P-S, and RFtest are $4.18 \times 10^{-2}$, $4.62 \times 10^{-1}$, $3.24 \times 10^{-1}$, $5.64 \times 10^{-2}$, $2.16 \times 10^{-1}$, $6.60 \times 10^{-2}$, and $7.47 \times 10^{-1}$, respectively. For the SBP indicator, the corresponding *p*-values are $2.15 \times 10^{-3}$, $1.02 \times 10^{-2}$, $6.32 \times 10^{-2}$, $1.01 \times 10^{-2}$, $9.70 \times 10^{-2}$, $1.11 \times 10^{-2}$, and $5.46 \times 10^{-2}$. For the DBP indicator, the *p*-values are $2.37 \times 10^{-2}$, $1.95 \times 10^{-1}$, $1.48 \times 10^{-1}$, $9.85 \times 10^{-2}$, $2.82 \times 10^{-1}$, $1.21 \times 10^{-1}$, and $1.64 \times 10^{-1}$. Based on a significance level of 0.05, only E-MANOVA detected a significant association for the BMI and DBP phenotypes. For the SBP phenotype, E-MANOVA, MIATDS, MiRKAT, and P-S all showed significant associations, and E-MANOVA again generated the most significant result with the smallest *p*-value of $2.15 \times 10^{-3}$.

To further demonstrate that our method can lead to new discoveries, we performed a deeper analysis of the dataset GhensiP_2019 [45]. This dataset contains case-control data for the disease mucositis with 84 samples. Mucositis is an inflammation and ulceration of the mucous membranes lining the digestive tract, commonly affecting the mouth and throat. The condition affects over 50% of patients with dental implants (a common method to restore missing teeth since the late seventies), leading in most cases to implant loosening or removal. We aimed to investigate the link between the plaque microbiome and mucositis within the GhensiP_2019 dataset. The *p*-values obtained from all seven methods were as follows: E-MANOVA ($1.02 \times 10^{-3}$), MIATDS ($1.63 \times 10^{-2}$), MiHC ($8.55 \times 10^{-3}$), MiRKAT ($2.13 \times 10^{-3}$), MiSPU ($4.21 \times 10^{-2}$), P-S ($1.40 \times 10^{-3}$), and RFtest ($2.15 \times 10^{-2}$). Only our proposed method, E-MANOVA, reached the Bonferroni-corrected significance level of $\alpha \approx 1.35 \times 10^{-3}$. This indicated that, under this stringent correction, all other competing methods would have failed to detect a significant correlation between mucositis and the plaque microbiome, a conclusion that would be inconsistent with the results of subsequent biological analyses.

**Fig. 4:**
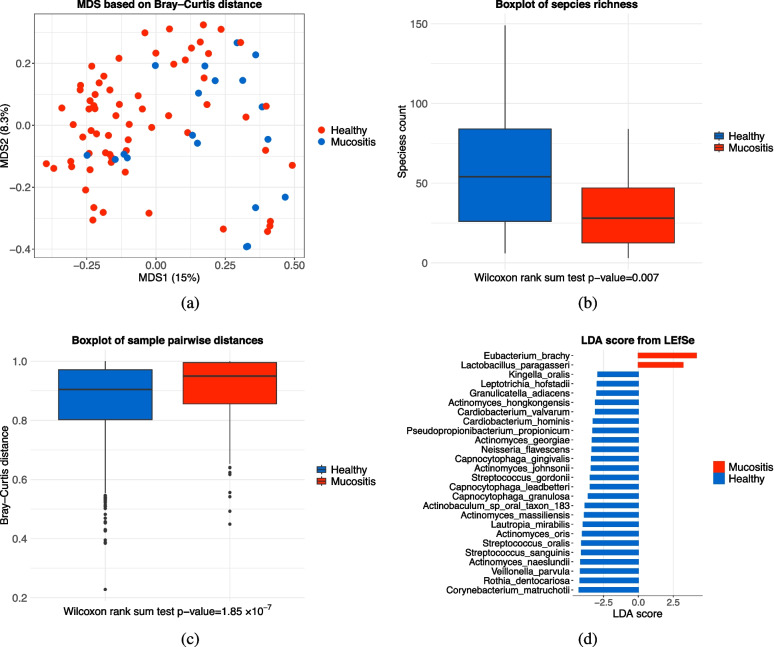
**a** Multidimensional scaling (MDS) ordination plot of healthy and mucositis samples based on the Bray–Curtis distance highlights a strong condition-specific clustering. **b** Alpha-diversity estimated as the richness of species. *P*-value was obtained by the two-tailed Wilcoxon rank sum test. **c** Intra-condition beta-diversity using Bray–Curtis distance. *P*-value was obtained by the two-tailed Wilcoxon rank sum test. **d** LDA score from Linear discriminant analysis Effect Size(LEfse) for microbiome species reach significance level 0.01 with Kruskal-Wallis rank sum test

Figure 4a shows the Multidimensional Scaling (MDS) ordination plot of healthy and mucositis samples based on the Bray–Curtis distance, which highlights a strong condition-specific clustering. Figure 4b and c use boxplots to show the difference in species richness (alpha diversity) and pairwise Bray–Curtis distance (beta diversity) between the healthy and mucositis groups. The *p*-values from the Wilcoxon rank sum test are 0.007 and $1.85 \times 10^{-7}$, respectively, indicating that there is a significant difference between the healthy and mucositis groups in terms of both alpha and beta diversity. Finally, Fig. 4d shows the LDA scores of 26 species reaching a significance level of 0.01 with the Kruskal-Wallis rank sum test. The absolute values of these LDA scores are all greater than 2.5, indicating that their relative abundances show extremely significant differences between groups and possess strong discriminatory power to distinguish between the different sample groups. We also conducted a Wilcoxon rank sum test on all species, and the smallest *p*-value was generated by the species *Capnocytophaga gingivalis*, with a *p*-value of $1.36 \times 10^{-4}$. *Capnocytophaga gingivalis* was also reported in another article to characterize the complex and diverse healthy plaque [45]. These facts indicate that there is a significant correlation between mucositis and the plaque microbiome that requires further study. Our proposed method E-MANOVA successfully detected these associations where other methods might lead to false negative results.

## Discussion

In this study, we introduced E-MANOVA, a novel ensemble method, to test the association with microbiome data, thereby addressing several limitations in traditional PERMANOVA-based analyses. Firstly, it has been shown that the null distribution of PERMANOVA-based test statistics is closely related to a mixture of chi-square distributions. Given the heavy-tailed nature of these distributions, our method involves raising the matrix $\boldsymbol{HSH}$ to the *r*th power, which adjusts the weights of the chi-square components, thus enhancing the power and robustness of our proposed method compared to existing ones. Secondly, we directly calculate the moments and use the Pearson type III distribution to approximate the null distribution, thus eliminating the computationally expensive permutation procedures. Our proposed method can generate arbitrarily small *p*-values without adding any computational burden. Thirdly, while PERMANOVA methods allow for the use of multiple distances, determining the most effective distance for optimal results remains challenging. Through simulations, we demonstrated that different distances exhibit varied performances in diverse scenarios. Our approach leverages the Cauchy combination method, effectively integrating results from multiple distances to construct a robust statistic without requiring permutation procedures.

We also note that while E-MANOVA is an association test, it is fully compatible with the structure used for Principal Coordinates Analysis (PCoA). The PCoA plot, a critical visualization tool in microbiome research, is also generated from the eigendecomposition of the same fundamental centered similarity matrix, $\boldsymbol{HSH}$, that our method utilizes. Therefore, researchers can and should continue to use PCoA to visualize the distance matrix that serves as the input for E-MANOVA, just as one would with PERMANOVA. We have demonstrated this practice in our own manuscript (e.g., Fig. 4a). Furthermore, our method provides a novel framework that enhances the interpretation of this PCoA. The standard PERMANOVA statistic ($T_0$, which is $T_{d,r}$ when $r=1$) implicitly weights all eigenvector directions (the principal coordinates) by their corresponding eigenvalues. Our E-MANOVA method, by applying the power *r* (i.e., calculating $(\boldsymbol{HSH})^r$), explicitly re-weights the contribution of these eigenvectors. A small *r* (e.g., $r=0.125$) up-weights the contribution of eigenvectors with small eigenvalues (i.e., higher-order principal coordinates), while a large *r* (e.g., $r=2$) focuses the test’s power on the dominant eigenvectors (i.e., the first few PCoA axes). By examining the component *p*-values for different *r* values, a researcher can gain additional insight into which scale of variation (i.e., which set of principal coordinates) best captures the association signal.

While theoretical connections between the null distribution of the PERMANOVA pseudo-*F* statistic and a mixture of chi-square distributions have been established, we still used moment approximation to approximate the null distribution. The main reason is that this proof crucially relies on the condition that the distance used in PERMANOVA generates a positive semi-definite (PSD) kernel [25]. This is guaranteed for Euclidean distance but not proven for distances commonly used in microbiome data such as weighted UniFrac distances, unweighted UniFrac distances, and Bray-Curtis distance. In our simulation, negative eigenvalues of the kernel consistently occurred, indicating that these distance measures do not produce a PSD kernel.

Our proposed statistical framework offers flexibility in its configuration. In this study, we employed weighted UniFrac, Unweighted UniFrac, and Bray-Curtis distances due to their widespread usage, covering scenarios both with and without phylogenetic tree information. Other distances like Jaccard distance may also be used. The choice of the set of *r* values, *R*, is also crucial. A set that is too dense could potentially dilute statistical power when using the Cauchy combination method. In this study, we selected a geometric sequence $R=\{0.125, 0.25, 0.5, 1, 2\}$. This choice is justified for two primary reasons. First, the inclusion of $r=1$ is theoretically important, as the base statistic with $r=1$ is equivalent to the traditional PERMANOVA statistic. This ensures that E-MANOVA formally incorporates the classic test as a special case, enhancing its robustness. Second, a geometric sequence is an effective strategy for exploring parameters that operate on a multiplicative scale, analogous to the common practice of using a log-scaled grid for tuning parameters in regularization methods [71]. To validate this choice against a sparser set (e.g., $R=\{0.125, 0.5, 2\}$), we conducted additional simulations (see Supplemental Material Section 3). The results demonstrated that the empirical power was remarkably similar between the two sets, indicating that the test is robust to the inclusion of these intermediate values. Given this robustness, and the strong justification for including $r=1$, we retained our proposed set. Other settings of *r* may also be used.

PERMANOVA-based methods have diverse applications in fields such as genetics [27, 72], genomics [73], and neurosciences [74], indicating the potential for generalizing our proposed method to these varied domains. Although some theoretical studies exist [25], their conclusions cannot be directly generalized to microbiome data. This is because the distances used in microbiome data may not lead to a positive-semi definite kernel. Thus, the theoretical properties of PERMANOVA-based methods using different kinds of distances still require further investigation. Additionally, although PERMANOVA methods do not impose restrictions on the number of OTUs considered, it is vital to ensure that the number of predictors does not exceed the sample size. Further studies are needed to extend PERMANOVA-based methods to high-dimensional settings where the number of predictor variables exceeds the sample size.

## Supplementary Information

Supplementary Material 1.

## Data Availability

The real data of cigarette smokers is published in [12] and can be freely and openly accessed in R package *GUniFrac*. For detailed instructions regarding the installation of this package and the downloading of data, please visit https://cran.r-project.org/web/packages/GUniFrac/index.html. The real data of curated microbiome data is published in [36] and can be freely and openly accessed in R package *curatedMetagenomicData*. For detailed instructions regarding the installation of this package and the downloading of relevant data, please visit https://bioconductor.org/packages/release/data/experiment/vignettes/curatedMetagenomicData/inst/doc/curatedMetagenomicData.html. The corresponding code for E-MANOVA is available at https://github.com/amss-stat/E-MANOVA and can be directly installed in R.
