# Supplementary material for: Ensemble test for microbiome data
Source: Microbiome. 2026 Mar 10;14:118. doi: 10.1186/s40168-026-02367-z (PMC13088834; doi:10.1186/s40168-026-02367-z)
Supplement: Supplementary file 2 — Supplementary Material 1. [file 40168_2026_2367_MOESM1_ESM.pdf]

Supplemental Material on "Ensemble test for microbiome data"

Deliang Bu, Jingxin Yan, Wanshuo Yang, Xiaoyu Zhang, Qizhai Li

In this Supplemental Material, we provide the following results. Section 1 gives the theoretical proof for calculating the first three moments of $T_{d,r}$ under null hypothesis. In Section 2, the quantile-quantile plot is used to examine the approximate accuracy of Pearson type III distribution. In Section 3, we provide the simulation result for scenarios of binary phenotypes. Section 3 also provides an additional simulation study comparing the statistical power for different choices of the parameter set R.

1 P-value calculation of $T_{d,r}$

Normally, the permutation procedure is needed for PERMANOVA-based method, which is computationally expensive and generates different results run multiple times on the same dataset. This variability arises due to their reliance on generating random samples to compute permutation null distributions. E-MANOVA can completely avoid intensive computation procedure and generate the same result with the same data.

Recall that the E-MANOVA test statistics with fix d and r in main text section 2.3 is

$$T_{d,r} = tr\{(\boldsymbol{H}_{\boldsymbol{X}} - \boldsymbol{H}_{\boldsymbol{X}_2})(\boldsymbol{K}^*)^r\}.$$

It can further be rewritten in the form of

$$T_{d,r} = tr(\boldsymbol{A}\boldsymbol{W}),$$

where $\boldsymbol{A} = \boldsymbol{H}_{\boldsymbol{X}} - \boldsymbol{H}_{\boldsymbol{X}_2}$ and $\boldsymbol{W} = \boldsymbol{H}(\boldsymbol{K}^*)^r\boldsymbol{H}$. It is easily to be proven since $\boldsymbol{H}$ is a centering matrix and all the columns of $\boldsymbol{X}$ are already centered, thus $\boldsymbol{H}\boldsymbol{X} = \boldsymbol{X}$ and $\boldsymbol{H}\boldsymbol{X}_2 = \boldsymbol{X}_2$. To avoid the potential computational burden, we adopt an alternative strategy based on the result derived in[**?**] that can directly calculate the moments of permuted null distribution without generating its null distribution and approximating the empirical null distribution with a known distribution that matches its moments. The following lemma 1 proves the properties of matrix $\boldsymbol{W}$ and Theorem 1 establishes the first three moments of $T_{d,r}$ without using permutation.

Lemma 1: *Denote $\boldsymbol{E}_{ij}$, $i \neq j$ as a $n \times n$ matrix exchanging ith row and jth row of identity matrix $\boldsymbol{I}_n$. Then,*

$$\boldsymbol{W}\big|_{i\leftrightarrow j} = \boldsymbol{E}_{ij}\boldsymbol{W}\boldsymbol{E}_{ij},$$

here $\boldsymbol{W}\big|_{i\leftrightarrow j}$ *indicates* $\boldsymbol{W}$ *switching* i*th sample and* j*th samples.*

Proof: Since the elements in $\boldsymbol{S}$, denote as s_{ij}, represent the similarity between ith and jth subjects. Thus, it is easy to see that

$$\boldsymbol{S}\big|_{i\leftrightarrow j} = \boldsymbol{E}_{ij}\boldsymbol{S}\boldsymbol{E}_{ij}, i, j = 1, 2, \cdots, n.$$

Since $\boldsymbol{H}$ is a symmetric matrix, we have

$$\boldsymbol{H}\boldsymbol{E}_{ij}\boldsymbol{S}\boldsymbol{E}_{ij}\boldsymbol{H} = \boldsymbol{E}_{ij}\boldsymbol{H}\boldsymbol{S}\boldsymbol{H}\boldsymbol{E}_{ij}.$$

$\boldsymbol{HSH}$ can be decomposed as $\boldsymbol{HSH} = \boldsymbol{Q}\boldsymbol{\Lambda}\boldsymbol{Q}^\top$ where $\boldsymbol{Q}$ is an orthogonal matrix whose columns are the orthogonal eigenvectors of $\boldsymbol{HSH}$, and $\boldsymbol{\Lambda}$ is a diagonal matrix whose entries are the eigenvalues of $\boldsymbol{HSH}$. We have

$$\boldsymbol{E}_{ij}\boldsymbol{H}\boldsymbol{S}\boldsymbol{H}\boldsymbol{E}_{ij} = \boldsymbol{E}_{ij}\boldsymbol{Q}\boldsymbol{\Lambda}\boldsymbol{Q}^\top\boldsymbol{E}_{ij}.$$

The matrix $\boldsymbol{K}^*$ is obtained by $\boldsymbol{K}^* = \boldsymbol{Q}\boldsymbol{\Lambda}^*\boldsymbol{Q}^\top$, with $\boldsymbol{\Lambda}^* = diag(|\lambda_1|, |\lambda_2|, \cdots, |\lambda_n|)$. Thus, we can conclude that

$$\boldsymbol{K}^*_{i\leftrightarrow j} = \boldsymbol{E}_{ij}\boldsymbol{K}^*\boldsymbol{E}_{ij}.$$

Next, since $\boldsymbol{W} = \boldsymbol{H}(\boldsymbol{K}^*)^r\boldsymbol{H}$, we have

$$(\boldsymbol{K}^*_{i\leftrightarrow j})^r = (\boldsymbol{E}_{ij}\boldsymbol{K}^*\boldsymbol{E}_{ij})^r.$$

Then, we can write that

$$(\boldsymbol{E}_{ij}\boldsymbol{K}^*\boldsymbol{E}_{ij})^r = (\boldsymbol{E}_{ij}\boldsymbol{Q}\boldsymbol{\Lambda}^*\boldsymbol{Q}^\top\boldsymbol{E}_{ij})^r = ((\boldsymbol{E}_{ij}\boldsymbol{Q})\boldsymbol{\Lambda}^*(\boldsymbol{E}_{ij}\boldsymbol{Q}^\top))^r,$$

where $\boldsymbol{Q}$ is orthonormal, resulting in that $\boldsymbol{E}_{ij}\boldsymbol{Q}$ is the eigenvectors of $\boldsymbol{E}_{ij}\boldsymbol{K}^*\boldsymbol{E}_{ij}$. Thus, by the definition of the rth power of matrix, we can conclude that

$$(\boldsymbol{E}_{ij}\boldsymbol{K}^*\boldsymbol{E}_{ij})^r = (\boldsymbol{E}_{ij}\boldsymbol{Q}(\boldsymbol{\Lambda}^*)^r\boldsymbol{Q}^\top\boldsymbol{E}_{ij}).$$

Finally, we conclude the proof that

$$\boldsymbol{W}_{i\leftrightarrow j} = \boldsymbol{H}(\boldsymbol{E}_{ij}\boldsymbol{Q}(\boldsymbol{\Lambda}^*)^r\boldsymbol{Q}^\top\boldsymbol{E}_{ij})\boldsymbol{H} = \boldsymbol{E}_{ij}\boldsymbol{W}\boldsymbol{E}_{ij}.$$

The last step is because $\boldsymbol{H}$ is symmetric, thus $\boldsymbol{H}$ and $\boldsymbol{E}_{ij}$ are exchangeable.

Lemma 1 indicates switching ith sample with jth of sample of $\boldsymbol{W}$ is equal to exchanging both rows and columns i and j. With this propriety of $\boldsymbol{W}$, we can directly use the theorem 1 to calculate the first three moments of $T_{d,r}$ without permutation.

Theorem 1: *Let $\boldsymbol{A}$ be a symmetric matrix, with $\boldsymbol{A}\boldsymbol{1}_n = 0$, $\boldsymbol{W}$ is a symmetric matrix and satisfies the condition that switching ith sample with jth sample is equivalent to exchanging both rows and columns i and j of $\boldsymbol{W}$. Then the first three moments of the test statistics $T = tr(\boldsymbol{A}\boldsymbol{W})$ can be directly calculated in close form. Denote the first three moments of T considering all $n!$ permutations as $E_p(T)$, $Var_p(T)$, and $E_p(T^3)$, we have*

$$E_p(T) = \frac{\operatorname{tr}(\boldsymbol{A})\operatorname{tr}(\boldsymbol{W})}{n-1},$$

$$Var_p(T) = \frac{2((n-1)L_2 - L^2)((n-1)\widetilde{L}_2 - \widetilde{L}^2)}{(n-1)^2(n+1)(n-2)} \\ + \frac{(n(n+1)M_2 - (n-1)(L^2 + 2L_2))(n(n+1)\widetilde{M_2} - (n-1)(\widetilde{L}^2 + 2\widetilde{L}_2))}{(n+1)n(n-1)(n-2)(n-3)},$$

$$n(n-1)(n-2)(n-3)(n-4)(n-5)E_p(T^3) = n^2(n+1)(n^2+15n-4)M_3\widetilde{M_3} \\ + 4(n^4 - 8n^3 + 19n^2 - 4n - 16)U\widetilde{U} + 24(n^2 - n - 4)(U\widetilde{B} + B\widetilde{U}) + 6(n^4 - 8n^3 + 21n^2 - 6n - 24)B\widetilde{B} \\ + 12(n^4 - n^3 - 8n^2 + 36n - 48)R\widetilde{R} + 12(n^3 - 2n^2 + 9n - 12)(LM_2\widetilde{R} + R\widetilde{L}\widetilde{M_2}) \\ + 3(n^4 - 4n^3 - 2n^2 + 9n - 12)L\widetilde{L}M_2\widetilde{M_2} + 24\{(n^3 - 3n^2 - 2n + 8)(R\widetilde{U} + \widetilde{U}R) \\ + (n^3 - 2n^2 - 3n + 12)(R\widetilde{B} + B\widetilde{R})\} + 12(n^2 - n + 4)(LM_2\widetilde{U} + U\widetilde{L}\widetilde{M_2}) \\ + 6(2n^3 - 7n^2 - 3n + 12)(LM_2\widetilde{B} + B\widetilde{L}\widetilde{M_2}) - 2n(n-1)(n^2 - n + 4)\{(2U + 3B)\widetilde{M_3} + (2\widetilde{U} + 3\widetilde{B})M_3\} \\ - 3n(n-1)^2(n+4)\{(LM_2 + 4R)\widetilde{M_3} + (\widetilde{L}\widetilde{M_2} + 4\widetilde{R})M_3\} + 2n(n-1)(n-2)\{(L^3 + 6LL_2 + 8L_3)\widetilde{M_3} \\ + (\widetilde{L}^3 + 6\widetilde{L}\widetilde{L_2} + 8\widetilde{L_3})M_3\} + L^3((n^3 - 9n^2 + 23n - 14)\widetilde{L}^3 + 6(n-4)\widetilde{L}\widetilde{L_2} + 8\widetilde{L_3}) + 6LL_2((n-4)\widetilde{L}^3 \\ + (n^3 - 9n^2 + 24n - 14)\widetilde{L}\widetilde{L}_2 + 4(n-3)\widetilde{L_3}) + 8L_3(\widetilde{L}^3 + 3(n-3)\widetilde{L}\widetilde{L}_2 + (n^3 - 9n^2 + 26n - 22)\widetilde{L_3}) \\ - 16(L^3\widetilde{U} + U\widetilde{L}^3) - 6(LL_2\widetilde{U} + U\widetilde{L}\widetilde{L_2})(2n^2 - 10n + 16) - 8(L_3\widetilde{U} + U\widetilde{L_3})(3n^2 - 15n + 16) \\ - (L^3\widetilde{B} + B\widetilde{T}^3)(6n^2 - 30n + 24) - 6(LL_2\widetilde{B} + B\widetilde{L}\widetilde{L_2})(4n^2 - 20n + 24) \\ - 8(L_3\widetilde{B} + B\widetilde{L_3})(3n^2 - 15n + 24) - (n-2)\{24(L^3\widetilde{R} + R\widetilde{L}^3) + 6(LL_2\widetilde{R} + R\widetilde{L}\widetilde{L_2})(2n^2 - 10n + 24) \\ + 8(L_3\widetilde{R} + R\widetilde{L_3})(3n^2 - 15n + 24) + (3n^2 - 15n + 6)(L^3\widetilde{L}\widetilde{M_2} + LM_2\widetilde{L}^3) \\ + 6(LL_2\widetilde{L}\widetilde{M_2} + LM_2\widetilde{L}\widetilde{L_2})(n^2 - 5n + 6) + 48(L_3\widetilde{L}\widetilde{M_2} + LM_2\widetilde{L_3})\},$$

where $L = tr(\boldsymbol{A})$, $L_2 = tr(\boldsymbol{A}^2)$, $L_3 = tr(\boldsymbol{A}^3)$, $\widetilde{L} = tr(\boldsymbol{W})$, $\widetilde{L}_2 = tr(\boldsymbol{W}^2)$, $\widetilde{L}_3 = tr(\boldsymbol{W}^3)$, $M_2 = \sum_{i=1}^n a_{ii}^2$, $M_3 = \sum_{i=1}^n a_{ii}^3$, $\widetilde{M}_2 = \sum_{i=1}^n w_{ii}^2$, $\widetilde{M}_3 = \sum_{i=1}^n w_{ii}^3$, $U = \sum_{i=1}^n \sum_{i=1}^n a_{ij}^3$, $\widetilde{U} = \sum_{i=1}^n \sum_{i=1}^n w_{ij}^3$, $R = (diag(\boldsymbol{A}))^\top (diag(\boldsymbol{A}^2))$, $B = (diag(\boldsymbol{A}))^\top \boldsymbol{A}(diag(\boldsymbol{A}))$, $\widetilde{R} = (diag(\boldsymbol{W}))^\top (diag(\boldsymbol{W})^2)$, $\widetilde{B} = (diag(\boldsymbol{W}))^\top \boldsymbol{W}(diag(\boldsymbol{W}))$.

The proof of theorem 1 is similar to [1] after rewriting $T_{d,r}$ as $T_{d,r} = tr(\boldsymbol{AW})$, where $\boldsymbol{A}$ and $\boldsymbol{W}$ satisfy the condition of theorem 1. We can then calculate the close from expression of the three moments of $T_{d,r}$ without using any permutation procedure.

2 Approximation accuracy

In this section, we use quantile-quantile plots to demonstrate the approximation of the Pearson type III distribution for $T_{d,r}$ with different values of d and r. As shown in the figures, the approximation performs well across different values of d and r.

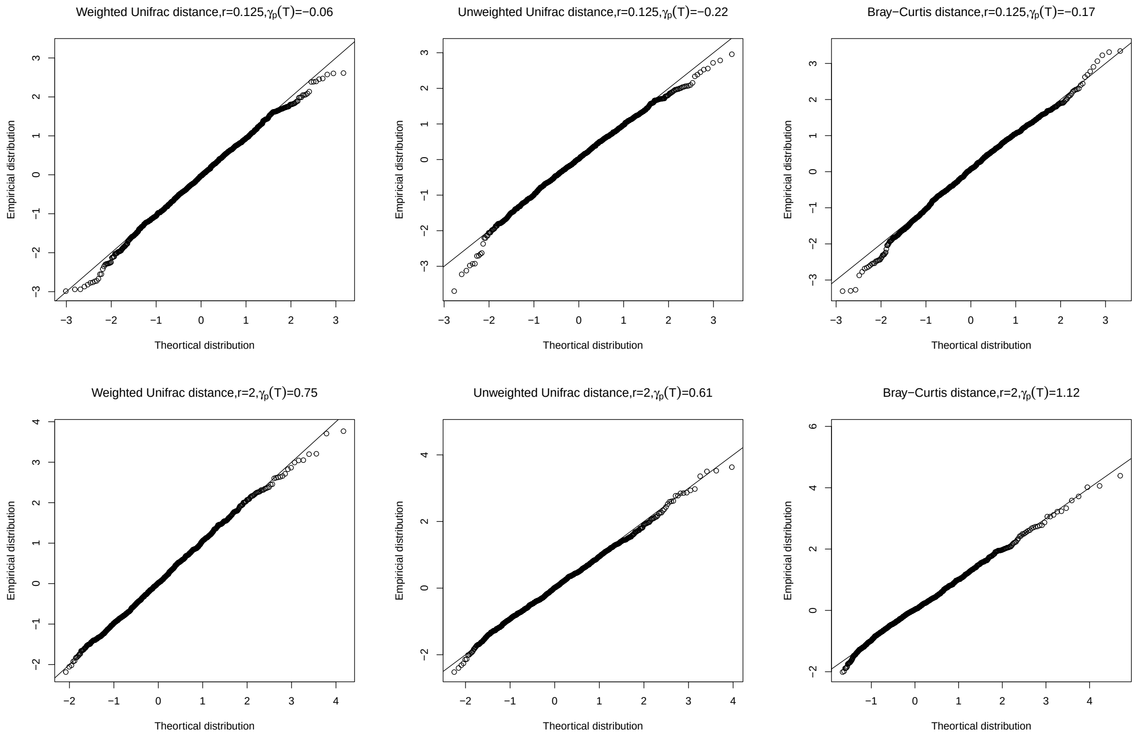

Figure 1: The empirical distribution of $T_{d,r}$ versus the theoretical Pearson type III distribution with parameters estimated based on the method in Section 1. Different scenarios were considered with a continuous outcome variable, $r = 0.125, 2$ and $d = 1, 2, 3$ represent three different distances (weighted UniFrac distance, Unweighted UniFrac distance and Bray-Curtis distance). All quantile-quantile plots are drawn based on 1000 random samples.

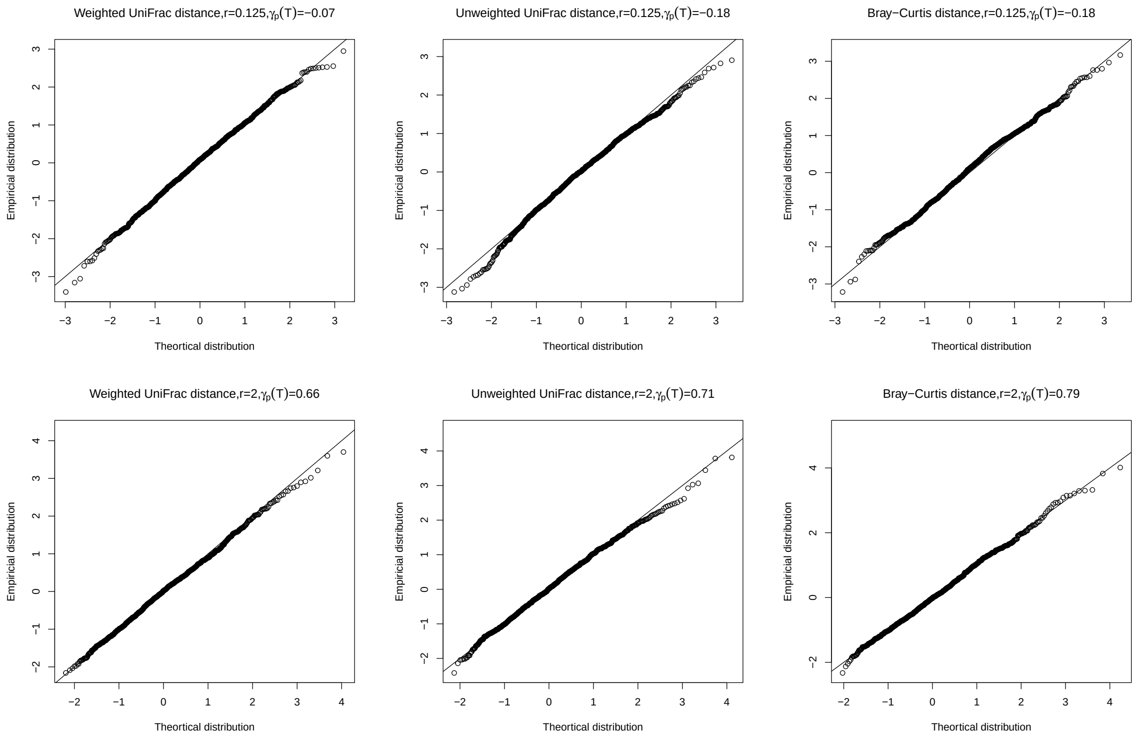

Figure 2: The empirical distribution of $T_{d,r}$ versus the theoretical Pearson type III distribution with parameters estimated based on the method in Section 1. Different scenarios were considered with a binary outcome variable, $r = 0.125, 2$ and $d = 1, 2, 3$ represent three different distances (weighted UniFrac distance, Unweighted UniFrac distance and Bray-Curtis distance). All quantile-quantile plots are drawn based on 1000 random samples.

3 Additional simulation results

This section provides additional simulation results focusing on a binary phenotype. The simulation strategy is outlined in the main text Methods section. Table 1 presents the empirical type I error rates, while empirical power results are depicted in Figure 3. similar results are observed as those for continuous phenotypes.

Table 1: Type I error rates of binary phenotypes with significance level $\alpha = 0.05$.

		E-MANOVA	MIATDS	MiHC	MiRKAT	MiSPU	P-S	RFtest
S1, Independent covariates	$\alpha = 0.05$	0.059	0.045	0.028	0.053	0.050	0.050	0.042
S1, correlated covariates	$\alpha = 0.05$	0.054	0.046	0.031	0.052	0.045	0.048	0.040
S2, Independent covariates	$\alpha = 0.05$	0.055	0.040	0.016	0.048	0.063	0.046	0.040
S2, correlated covariates	$\alpha = 0.05$	0.050	0.036	0.024	0.048	0.053	0.050	0.052
S3, Independent covariates	$\alpha = 0.05$	0.044	0.032	0.018	0.037	0.039	0.038	0.035
S3, correlated covariates	$\alpha = 0.05$	0.053	0.038	0.019	0.046	0.047	0.042	0.040
S4, Independent covariates	$\alpha = 0.05$	0.055	0.040	0.016	0.048	0.063	0.046	0.041
S4, correlated covariates	$\alpha = 0.05$	0.050	0.036	0.024	0.048	0.053	0.050	0.055

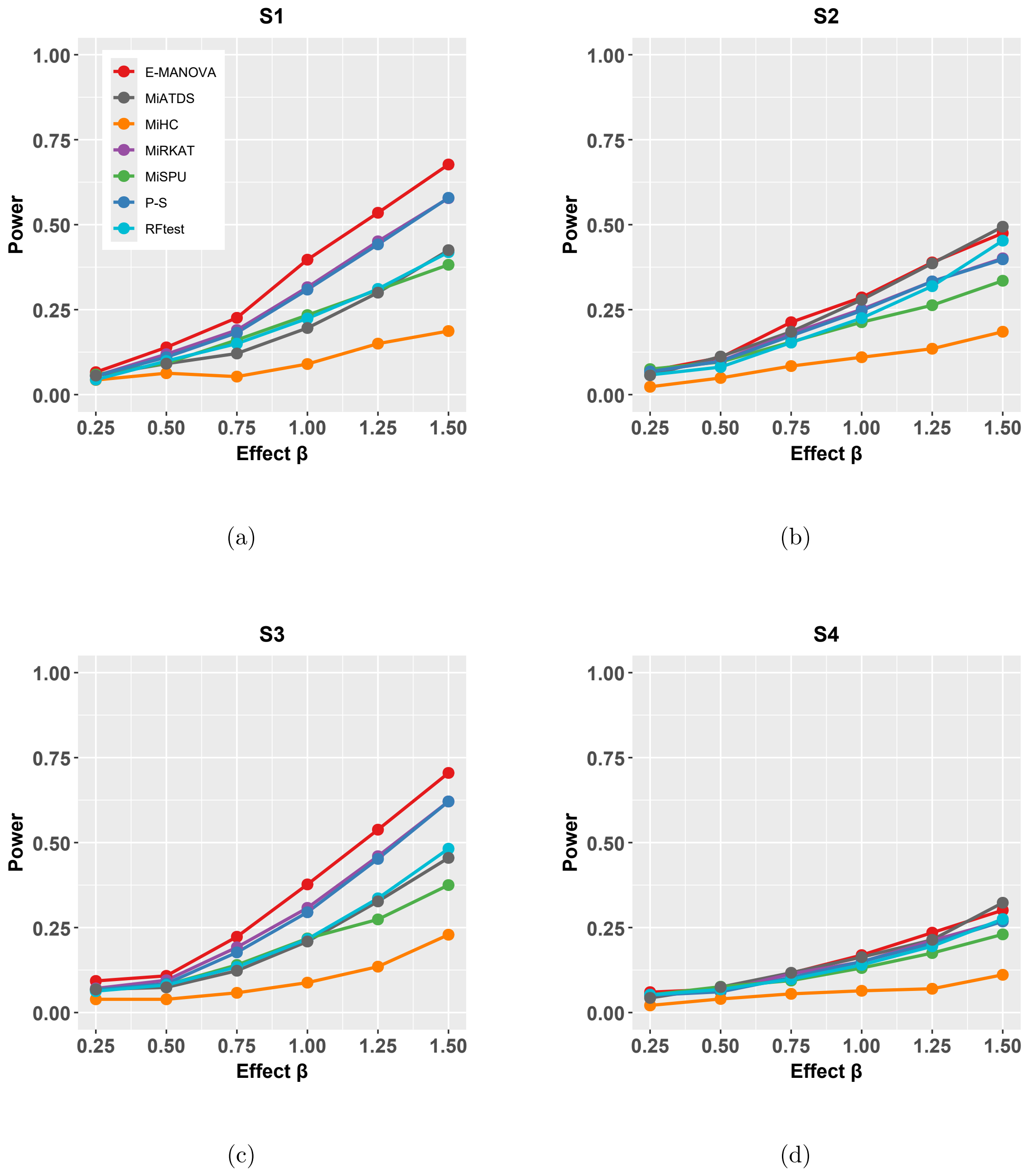

Figure 3: Empirical powers of E-MANOVA, MIATDS, MiHC, MiRKAT, MiSPU, P-S, and RFtest with binary phenotypes and independent covariates at significance level $\alpha = 0.05$.

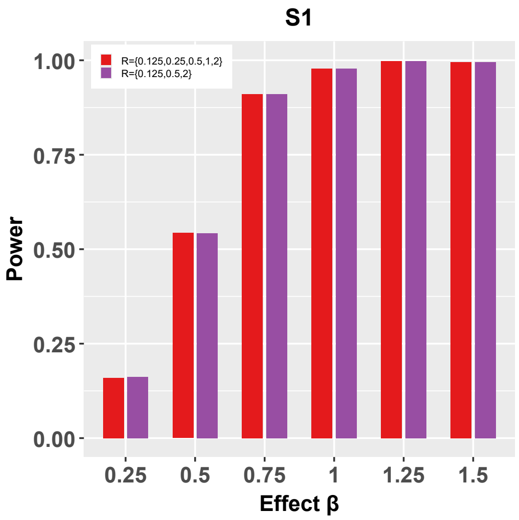

(a)

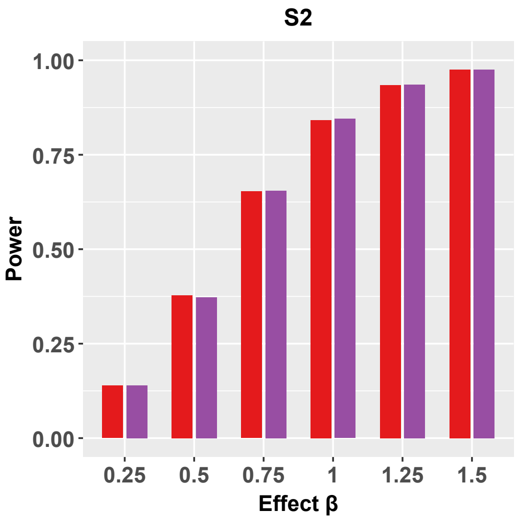

(b)

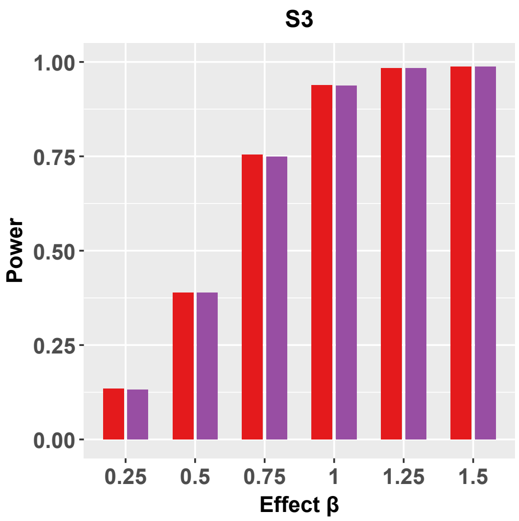

(c)

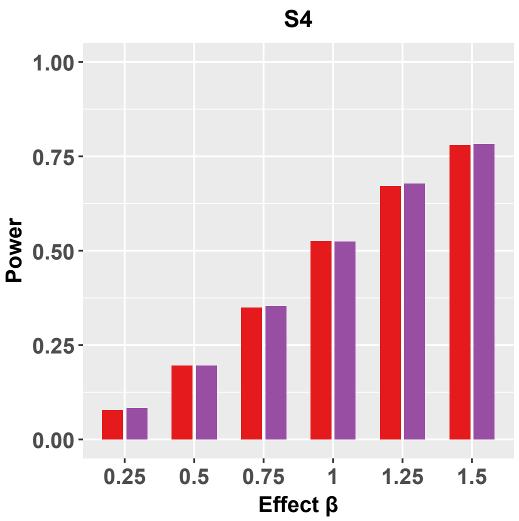

(d)

Figure 4: Empirical powers of E-MANOVA with parameter settings $R = \{0.125, 0.25, 0.5, 1, 2\}$ compare with $R = \{0.125, 0.5, 2\}$ with continuous phenotypes and independent covariates under scenarios $S1$ to $S4$ with significance level 0.05.

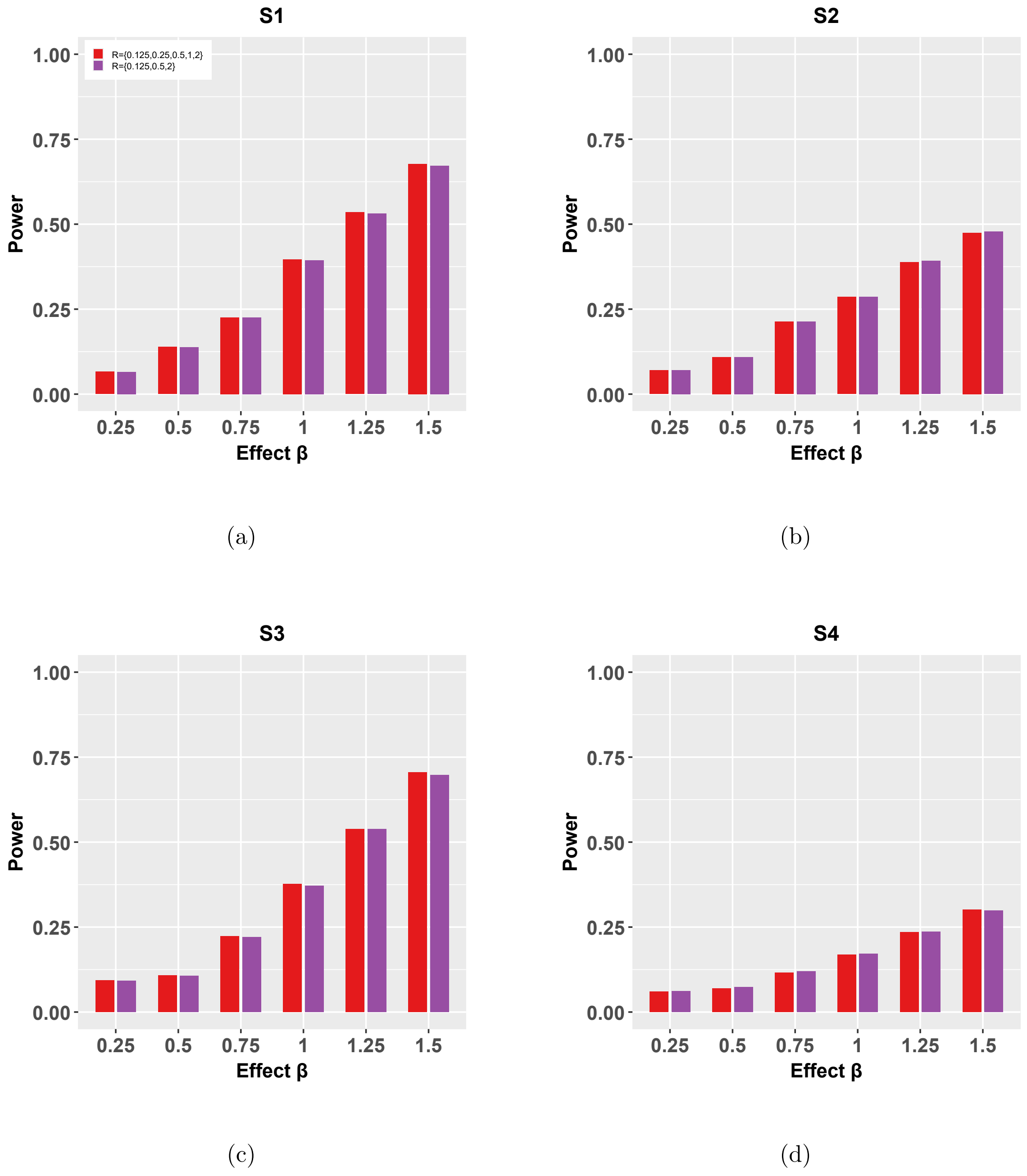

Figure 5: Empirical powers of E-MANOVA with parameter settings $R = \{0.125, 0.25, 0.5, 1, 2\}$ compare with $R = \{0.125, 0.5, 2\}$ with binary phenotypes and independent covariates under scenarios $S1$ to $S4$ with significance level 0.05.

Reference

1. Kazi-Aoual, F., Hitier, S., Sabatier, R., D. et al. (1995). Refined approximations to permutation tests for multivariate inference. *Computational Statistics & Data Analysis*, 20(6), 643–656.
